# ZNF395 Is an Activator of a Subset of IFN-Stimulated Genes

**DOI:** 10.1155/2017/1248201

**Published:** 2017-02-21

**Authors:** Linda Schroeder, Christine Herwartz, Darko Jordanovski, Gertrud Steger

**Affiliations:** Institute of Virology, University of Cologne, Fürst-Pückler-Strasse 56, 50935 Cologne, Germany

## Abstract

Activation of the interferon (IFN) pathway in response to infection with pathogens results in the induction of IFN-stimulated genes (ISGs) including proinflammatory cytokines, which mount the proper antiviral immune response. However, aberrant expression of these genes is pathogenic to the host. In addition to IFN-induced transcription factors non-IFN-regulated factors contribute to the transcriptional control of ISGs. Here, we show by genome wide expression analysis, siRNA-mediated suppression and Doxycycline-induced overexpression that the cellular transcription factor ZNF395 activates a subset of ISGs including the chemokines CXCL10 and CXCL11 in keratinocytes. We found that ZNF395 acts independently of IFN but enhances the IFN-induced expression of CXCL10 and CXCL11. Luciferase reporter assays revealed a requirement of intact NF*κ*B-binding sites for ZNF395 to stimulate the CXCL10 promoter. The transcriptional activation of CXCL10 and CXCL11 by ZNF395 was abolished after inhibition of IKK by BMS-345541, which increased the stability of ZNF395. ZNF395 encodes at least two motifs that mediate the enhanced degradation of ZNF395 in response to IKK activation. Thus, IKK is required for ZNF395-mediated activation of transcription and enhances its turn-over to keep the activity of ZNF395 low. Our results support a previously unrecognized role of ZNF395 in the innate immune response and inflammation.

## 1. Introduction

The interferon- (IFN-) mediated innate immune response is the first line defense against invading pathogens. IFNs limit the spread of infectious agents and stimulate the adaptive immune system by promoting the development of high affinity specific T and B cell responses and immunological memory (reviewed in [1]). The detection of pathogens by pattern recognition receptors results in the activation of three major downstream signaling pathways, the NF*κ*B, MAPKs, and IFN regulatory factor (IRF) pathway, which cooperate in the transcriptional activation of the expression of type I and type II IFNs and proinflammatory cytokines. Secreted type I IFNs bind to the ubiquitously expressed IFNA receptor (IFNAR) on the same cells as well as on neighboring cells and initiate a signaling cascade finally leading to the activation and phosphorylation of the signal transducers and activators of transcription (STAT) STAT1 and STAT2. This enables complex formation with IRF9 to build the trimeric transcription factor ISGF3 which binds to the IFN-stimulated response element (ISRE), present in the upstream region of ISGs. ISGF3 activates the transcription of a set of several hundred ISRE driven ISGs which have antiviral and proinflammatory activity. The products of ISGs counteract viral replication, transcription, and translation and thus establish an antiviral status in the infected and uninfected bystander cells and stimulate the adaptive immune response. Shortly after the IFN exposure cells acquire an IFN-desensitized state allowing recovery from the effects of IFN. Dysregulation of IFN production and signaling results in autoimmune disorders such as systemic lupus erythematosus (reviewed in [1]).

While the components of this canonical type I IFN signaling pathway are widely expressed, the production of type II IFN*γ*, which signals through the IFNG receptor (IFNGR), is largely restricted to the cells of the immune system. However, since the IFNGR is widely expressed nearly every cell can respond to IFN*γ*. The binding of IFN*γ* to the IFNGR results in the tyrosine phosphorylation of STAT1 monomers and the formation of STAT1:STAT1 homodimers, which translocate into the nucleus and bind to *γ*-activated sequence (GAS) elements present in the promoters of IFN*γ* responsive genes (reviewed in [2]). IFN*γ* has a profound role in mounting the cellular immune response and preferentially mediates an inflammatory response.

Types I and II IFN-mediated induction of proinflammatory mediators, including the chemokines CXCL9, CXCL10, and CXCL11, plays a key role in establishing local inflammation. The three chemokines are ligands of the CXCR3 receptor that is expressed on different leucocyte subsets including monocytes, natural killer (NK) cells, and activated T lymphocytes and attract these leucocytes to local sites of inflammation [3]. Signaling induced by binding of these chemokines to the CXCR3 controls the development and function of CD4^+^ T cell subsets and thus contributes to the regulation of the inflammatory process [4]. The CXCR3 receptor and its ligands, specifically CXCL10 and CXCL11, are also expressed on/from nonimmune cells, including skin keratinocytes and fibroblasts (reviewed in [5]). Here they have a functional role in late stages of wound healing [6] and they are involved in development of proinflammatory skin diseases such as the autoimmune disorder vitiligo [7, 8].

In addition to IFN, ISG expression is further regulated at several levels, including epigenetic factors, coactivators, and corepressors interacting with the phosphorylated STATs, chromatin-modifying complexes such as HDAC1, and other DNA binding transcriptional activators and repressors. For instance, many ISGs contain NF*κ*B binding motifs within their upstream regulatory region and STAT factors were found to cooperate with NF*κ*B. The transcriptional activity of NF*κ*B is controlled by the I*κ*B kinase (IKK) complex, which is activated upon signaling induced by multiple pattern recognition receptors and proinflammatory cytokines (reviewed in [9]).

Previously, we could show that the barely characterized transcription factor ZNF395 (also known as Papillomavirus Binding Factor [10] and Huntington's Disease Binding Protein HDBP2 [11]) modulates the IFN*α*-mediated induction of IFIT1, IFIT2, IFI16, and IFI44 [12]. ZNF395 is conserved to two other proteins, which are the SLC2A4 regulator (SLC2A4RG, also known as Glut4 enhancer binding protein (GLUT4EF) or HDBP1) and ZNF704 (also known as glucocorticoid induced gene 1 (GIG1)). ZNF395, SLC2A4RG, and ZNF704 share three highly conserved regions (CR1-3). The CR3 codes for a so-called cysteine clamp (C-clamp), which is also present in the e-tail isoforms of the vertebrate HMG-domain containing T Cell Factor/Lymphoid Enhancer Factor (TCF/LEF) transcription factors TCF1E and TCF4E. The C-clamp is a DNA binding zinc finger domain that is characterized by four highly conserved cysteines [13–15]. In TCF1E and TCF4E, the C-clamp acts as a second DNA binding domain that recognizes a DNA motif with the sequence RCGG (R = purine), also known as the helper site [16]. DNA binding of ZNF395 and SLC2A4RG is solely conferred by the C-clamp. DNA binding of the C-clamp of ZNF395 requires a sequence CCGG in vitro which is thus similar to the helper side [11, 17].

ZNF395 is ubiquitously expressed and little is known about its biological activity. Initially, we identified ZNF395 in keratinocytes by its ability to bind to and repress the HPV8 promoter which depended on the recruitment of the Sin3A/HDAC1/2 corepressor complex via direct interaction of ZNF395 with several components of this complex [17]. Several reports support a role of ZNF395 in carcinogenesis. ZNF395 was suggested to be involved in the suppression of metastasis, migration, and invasion [18, 19]. On the other hand functional studies and data based on genome wide expression analysis found increased ZNF395 expression in various cancer tissues and are in line with a role of ZNF395 supporting cancer progression [20–26]. Its overexpression in cancer tissues may partially be based on the fact that ZNF395 is a hypoxia induced gene [27, 28]. We could show that ZNF395 is a direct target gene of the hypoxia inducible factors HIF1*α* and HIF2*α*, the master regulators of the response to hypoxia [12, 29, 30]. ZNF395 was required for the maximal hypoxic induction of proinflammatory cytokines [30] indicating that ZNF395 is associated with hypoxia-associated inflammation, which is known to promote malignant progression [31].

Genome wide expression analysis further implicate a role of ZNF395 in the antiviral innate immune response. The factor was found to be downregulated upon high HIV1 loads and the peak replication of CMV in CD4^+^ and CD8^+^ T lymphocytes [32–34] and in CD8^+^ T cells in acute infectious mononucleosis patients compared to EBV infected patients in convalescence [35]. This motivated us to further characterize the contribution of ZNF395 to the IFN-regulated gene expression.

## 2. Materials and Methods

### 2.1. Cell Culture

Primary normal human keratinocytes (NHEK) were obtained from Promocell and cultivated in KGM2 medium. The immortalized keratinocyte cell line RTS3b [36] and the derived RTS3b-TR-FLAG-ZNF395-cell lines [12] were cultivated in E-Medium, the latter supplemented with Blasticidin and Zeocin. RTS3b-TR-FLAG-ZNF395 lines 1 and 2 were generated by the transfection of 1 *μ*g or 2 *μ*g pcDNA-TO-FLAGZNF395 expression vector followed by selection with Blasticidin and Zeocin. RTS3b-TR-FLAG-ZNF395-2 allowed higher expression of ZNF395. To induce the expression of ZNF395 the cells were incubated in Doxycycline (Dox) containing medium (1 *μ*g/ml) for 24 h. Pooled primary human fibroblasts, isolated from foreskin (HFF), were cultivated in DMEM supplemented with 10% FCS and the antibiotics Streptomycin/Penicillin. PolyI:C (from Invivogen) was used at 10 ng/ml for 24 h, TNF*α* (from Cell Signaling) at 1 ng/ml for 24 h, and BMS-345541 (from Sigma Aldrich) at 5 *μ*M for 24 h [12], a concentration resulting in the inhibition of the catalytic subunits of IKK*β* and IKK*α* in vitro [37].

### 2.2. Plasmid DNAs

The CXCL10 promoter-luciferase constructs have been described in [38]. The expression vector for pcDNA-FLAG-ZNF395 was used in [17]. The deletion or point mutations in ZNF395 were introduced by site directed mutagenesis or cloning of appropriate PCR products into pcDNA-3.1-FLAG vector. HA-IKK*β* (Addgene Plasmid 15470) and HA-IKK*α* (Addgene Plasmid 15469) are published [39].

### 2.3. Small RNA Interference and IFN-Treatment, CXCL10 ELISA

Small interfering RNAs (siRNAs) were obtained as a pool of four annealed double stranded RNA oligonucleotides from Dharmacon (siZNF395: M-020387 and siControl: D-0012061420). Cells were seeded in six wells and transfected with 50 pmol siRNA using Lipofectamine RNAiMax (Invitrogen) one day later. As indicated, 850 U/ml IFN*γ* (Biomol) or 1000 U/ml IFN*α* (Biomol) was added 24 h or 42 h after transfection. The cells were harvested 48 h after transfection and total cellular RNA was isolated. The supernatant was assayed for CXCL10 using an CXCL10 ELISA kit from BioLegend.

### 2.4. Transient Transfections and Western Blots

Cotransfections of siRNA and CXCL10-Luc promoter constructs were done in 24 wells using 7.5 pmol siRNA, 62.5 ng luciferase reporter plasmid and Lipofectamine 2000 (Invitrogen) according to the manufacturer's instruction. 48 h later the cells were harvested in 100 mM KPO_4_, pH 7.8, 0.1% NP40, and 1 mM DTT and the luciferase activity was determined with Promega's GloMax® Multi Reader as described previously [12]. RTS3b cells were transfected with the appropriate expression vectors using Fugene HD (Roche Diagnostics). The preparation of cell extracts, Western Blots (WB), and coimmunoprecipitations were performed as described previously [12].

### 2.5. RT-PCR, Microarray

Total RNA was isolated by the NucleoSpin® RNA Mini Kit from Macherey-Nagel (Düren, Germany). C-DNA synthesis and hybridization to Affymetrix Exon 2.0 ST array was performed by the group of Professor Nürnberg (CCG, Cologne, Germany). The raw data were processed with the help of the Affymetrix Expression and Transcriptome analysis console. For quantitative RT-PCR, 2 *μ*g of RNA was reverse transcribed using random primer and the Go-Script Reverse Transcriptase (Promega) or the Maxima reverse transcriptase (Invitrogen). QRT-PCR was performed with the Go-Taq qPCR mastermix including Sybr green (Promega) and a Roche Light Cycler 480 (Roche Diagnostics). The sequences of the primers used for PCR are provided in Table 1. The expression of the various factors was normalized against the house keeping gene hypoxanthine guanine phosphoribosyltransferase (HPRT). The fold changes of the relative gene expression values for the various factors in relation to cells transfected with siControl were calculated by Δ-comparative threshold method [40].

### 2.6. Statistical Analysis

Significance of the differences was determined by the *t*-test for paired samples.

## 3. Results

### 3.1. ZNF395 Regulates a Subset of ISGs in Response to IFN*α*

**Figure 1:**
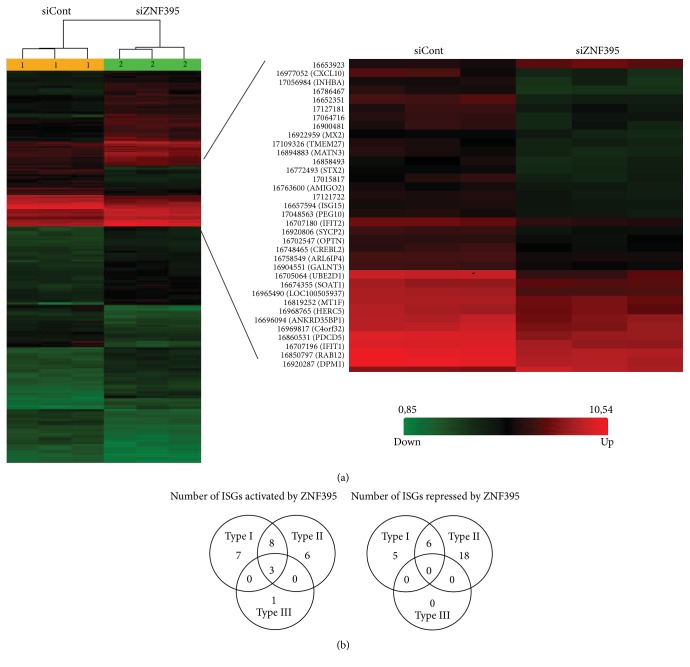
ZNF395 affects IFN*α*-mediated gene expression in keratinocytes. (a) Hierarchical clustering of transcriptome analysis of IFN*α* treated RTS3b cells, transfected with pooled siRNAs against ZNF395 (2, right) or control siRNA (1, left) in triplicate, using Affymetrix Hu Gene Chips. The gene clusters are shown on the right. In total, 156 genes were differentially expressed (*n* > 2-fold, *p* < 0.01). (b) Venn diagram. Among the differentially expressed genes 54 were IFN-regulated genes, with 25 genes being upregulated and 29 genes being repressed by ZNF395. The Venn diagram shows the number of type I, type II, and type III interferon regulated genes that are affected by ZNF395 in IFN*α* treated RTS3b cells, as proposed by the Interferome database.

We systematically addressed the impact of ZNF395 in the IFN*α*-mediated gene expression. Since we initially cloned ZNF395 from a keratinocyte cell line [10] we investigated the differential gene expression in response to ZNF395 and IFN*α* in keratinocytes. Type I IFNs comprise IFN*α*, IFN*β*, IFN*ε*, IFN*κ*, and IFN*ω*. While nearly every cell is capable of producing IFN*α*/*β* the other type I IFNs display tissue specific expression. Primary keratinocytes constitutively express IFN*κ* [41]. In order to avoid interference between endogenous IFN*κ* and the induction of ISGs by exogenously added IFN*α* we chose the keratinocyte cell line RTS3b that does not have constitutive expressed IFN*κ*, since it is epigenetically silenced by methylation (F. Stubenrauch, personal communication). We performed a microarray to analyze the ZNF395-dependent gene expression profile of IFN*α* treated RTS3b keratinocytes. RTS3b cells were transfected with control siRNA or with a pool of four siRNAs targeting ZNF395 to suppress the endogenous level of ZNF395. 18 h later the cells were incubated in medium containing IFN*α* for 6 h and total cellular RNA was isolated and transcribed to c-DNA. The expression of ZNF395 was reduced by 80% (data not shown). C-DNAs were hybridized to Affymetrix HuGene 2.0 ST Arrays. We identified 304 transcript clusters/terms that were differentially expressed by more than 2-fold (*p* < 0.01) due to the lack of ZNF395 (Figure 1(a)). These included 156 genes, with 57 genes with decreased and 99 genes with increased expression upon suppression of ZNF395. To identify the IFN-regulated genes we submitted the gene list to the Interferome database (v2.01), which contains experimentally and statistically (*p* < 0.05 and fold change > 2) validated IFN-regulated genes. This revealed that our gene list contained 25 known human IFN-regulated genes that were activated and 29 that were repressed by ZNF395. As depicted by the Venn diagram in Figure 1(b) the majority of the genes with altered expression upon suppression of ZNF395 were IFN type I and type II regulated (Figure 1(b)). The genes that our screen found to be activated by ZNF395 included well known ISGs such as CXCL10, CXCL11, IFIT1, IFIT2, TRIM22, HERC5, MX2, and ISG15 (Table 2). The gene products for TRIM22, HERC5, MX2, and ISG15 have been identified as restriction factors against HIV1 [42–48]. IL-18, DDX39B (BAT1) and HLA-DR (data not shown) were among the genes repressed by ZNF395. IFIT1, IFIT2, and PEG10, which we already found in our previous screen, analyzing the differentially expressed genes due to Doxycycline (Dox) induced overexpression of ZNF395 in an RTS3b cell line, were among the activated genes as well. The expressions of IFI16, SAMD9, and IFI44, which were also identified in our previous screen as genes activated by overexpression of ZNF395 [12], were decreased here by 1.5-fold (IFI16, *p* = 0.019 and SAMD9, *p* = 0.0002) and 1.49-fold (IFI44, *p* = 0.001) upon the knock-down of ZNF395. This congruency of the data sets indicated the reliability and reproducibility of the results of the two microarrays, identifying differentially expressed genes due to ZNF395 overexpression, as reported in [12], and due to suppression of ZNF395 in IFN*α* treated cells, as reported here.

### 3.2. ZNF395 Modulates the IFN*α*-Mediated Induction of Several ISGs

**Figure 2:**
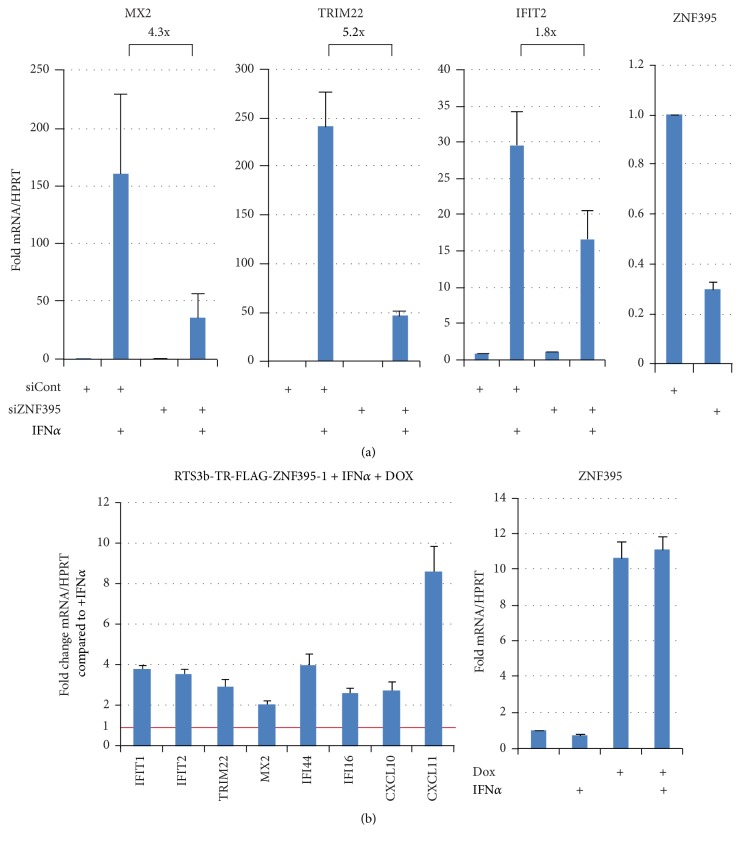
ZNF395 modulates the IFN*α*-mediated induction of ISGs. (a) QRT-PCR with RNA from RTS3b cells that have been transfected with pooled siRNA against ZNF395 or control siRNA, and treated with 1000 U/ml IFN*α* for 6 h as indicated. The RT-PCR results for MX2, TRIM22, and IFIT2 are shown. (b) The RTS3b-TR-FLAG-ZNF395-1 cell line, allowing the Dox-inducible overexpression of ZNF395, was treated with IFN*α*. The fold changes of the factors in response to the addition of Dox compared to minus Dox, which was set as 1 in all cases, are given. The expression of ZNF395 was monitored by qRT-PCR in RTS3b cells after transfection with pooled siRNA against ZNF395 or siControl and in the RTS3b-TR-FLAG-ZNF395-1 line, in the presence and absence of Dox. In all cases, the CP-values have been normalized against those for the house keeping gene HPRT. Fold changes were calculated by Δ-threshold method [40]. RT-PCR results are the means of three to six independent assays. Standard deviations are shown.

**Figure 3:**
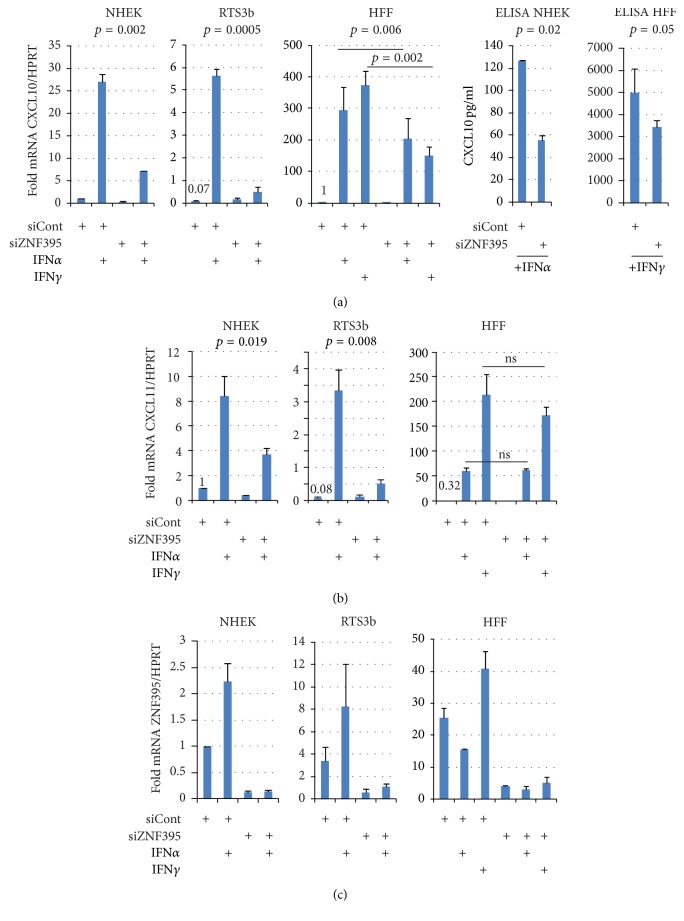
ZNF395 modulates the expression of CXCL10 and CXCL11. Primary human epithelial keratinocytes (NHEK), RTS3b cells, and primary human foreskin fibroblasts (HFF) were transfected with siRNA against ZNF395 or control siRNA and treated with IFN*α* or IFN*γ*, as indicated. (a) QRT-PCR was performed with primers to amplify CXCL10 and HPRT. The fold changes after normalization for the expression of HPRT were calculated and adjusted to the value of nontreated NHEK transfected with control siRNA, which was defined as 1. The result of ELISA-assays with the supernatant of NHEK and HFF to determine the concentrations of CXCL10 is shown on the right. (b) CXCL11 expression analyzed by qRT-PCR with RNA from siRNA transfected NHEK, RTS3b, and HFF treated with either IFN*α* or *γ*. (c) The relative expression level of ZNF395 in siControl transfected NHEK was set as 1 and the fold changes of all other cell types have been calculated. The diagrams show the means of three independent experiments. Standard deviations are given.

To further investigate the contribution of ZNF395 to the IFN-induced gene expression in more detail we selected specific genes requiring ZNF395 for their full activation by IFN*α*. We visualized the impact of ZNF395 by analyzing their IFN*α*-mediated induction after the suppression of ZNF395 by siRNA on the one hand (Figure 2(a)) and on the other hand after overexpression of ZNF395 in RTS3b-TR-FLAG-ZNF395-1 cells, containing a Dox-inducible expression vector for ZNF395, as described in [12] (Figure 2(b)). QRT-PCR revealed that siRNA transfection achieved a 70% knock-down of ZNF395 (Figure 2(a), see also Figure 3(c)) and the Dox-mediated induction of ZNF395 increased the amount of transcripts by 11-fold (Figure 2(b)). Thus, using these two approaches we were able to modulate the level of ZNF395 over a wide range.

The 160-fold activation of MX2 by IFN*α* dropped to 37-fold upon suppression of ZNF395, the 241-fold induction of TRIM22 was reduced to 46-fold, and IFN-induced stimulation of IFIT2 dropped from 30-fold to 16-fold in the absence of ZNF395 (Figure 2(a)). On the other hand, in the RTS3b-TR-FLAG-ZNF395-1 cell line, the Dox induced overexpression of ZNF395 further enhanced the IFN*α*-mediated activation of these ISGs, as well as of IFIT1, IFI16, IFI44, and CXCL10 by 2- to 4-fold. Strikingly, the IFN*α*-mediated transcription of CXCL11 was induced by 8.5-fold in the presence of Dox (Figure 2(b)). This is in line with the results of the microarray and shows that ZNF395 modulates the magnitude of the induction of several ISGs by IFN*α*.

### 3.3. ZNF395 Is Required for the Efficient IFN-Mediated Induction of CXCL10 in Primary Keratinocytes and Fibroblasts

We further focused on the role of ZNF395 in the control of the proinflammatory cytokines CXCL10 and CXCL11. The microarray found CXCL10 transcription as the most reduced following suppression of ZNF395 (Figure 1(a)). We addressed the role of ZNF395 in the expression of CXCR3 ligands in IFN*α* treated RTS3b cells, primary normal human epidermal keratinocytes (NHEK), and primary human foreskin fibroblasts (HFF). As before, all cell types were transiently transfected with a pool of siRNAs against ZNF395 to suppress ZNF395. To compare the expression of CXCL10/CXCL11 and ZNF395 among the different cell types, the gene specific qRT-PCR crossing points (CP) were normalized with those for the house keeping gene HPRT and the resulting values from untreated, siControl transfected NHEK were set as 1. This approach revealed that siControl transfected RTS3b cells had lower basal level of CXCL10 with 0.07 compared to primary keratinocytes (Figure 3(a)). The elevated amounts of CXCL10 in the NHEK may reflect the activity of endogenous IFN-*κ* in primary keratinocytes. IFN*α* increased the CXCL10 expression 27-fold in siControl but only 7-fold in siZNF395 transfected NHEK. In RTS3b, IFN*α* elevated CXCL10 mRNA level 75-fold in the control cells, and 7-fold when ZNF395 was suppressed, indicating that ZNF395 is required for the efficient induction of CXCL10 in both types of keratinocytes (Figure 3(a)). Fibroblasts are also strong producers of CXCR3 ligands [5]. Primary HFF had similar basal level of CXCL10 compared to NHEK but revealed a strong, 300-fold induction by IFN*α*. The suppression of ZNF395 decreased this to 200-fold. IFN*γ* stimulated CXCL10 by 400-fold in siControl and 150-fold in siZNF395-transfected HFF. Both differences were significant, indicating that ZNF395 is required for the maximal IFN*α* and *γ*-mediated induction of CXCL10 also in HFF. The decrease of the concentrations of CXCL10 produced by HFF and NHEK upon the suppression of ZNF395 could also be confirmed by an ELISA performed with the appropriate cell culture supernatants (Figure 3(a)).

We also analyzed the expression of CXCL11 under these conditions in the keratinocytes and fibroblasts. Although the net values differed, the si-RNA mediated suppression of ZNF395 significantly reduced the effects of IFN*α* in NHEK and RTS3b cells, indicating that ZNF395 is also required for the efficient IFN*α*-mediated induction of CXCL11 in keratinocytes (Figure 3(b)). Surprisingly, in HFF, the IFN*α* and IFN*γ*-mediated activations of CXCL11, which were much stronger compared to keratinocytes, did not significantly differ when ZNF395 was suppressed. Similarly, the transcription of CXCL9 was highly activated by both, IFN*α* and IFN*γ*, with no differences between siControl or siZNF395 transfected HFF (data not shown). Thus, it seems that ZNF395 is not significantly involved in the IFN-controlled transcription of CXCL9 and CXCL11 in HFF but in the IFN*α* and IFN*γ*-induced activations of CXCL10, while in keratinocytes it is required for CXCL11 as well. In addition, CXCL10 and CXCL11 are more efficiently activated by IFNs in HFF, which do not express IFN*κ* [49] and the contribution of ZNF395 seems to be less strong compared to keratinocytes.

### 3.4. Inhibition of IKK*β* by BMS-345541 Abolishes ZNF395-Mediated Activation of Endogenous CXCL10 and CXCL11

**Figure 4:**
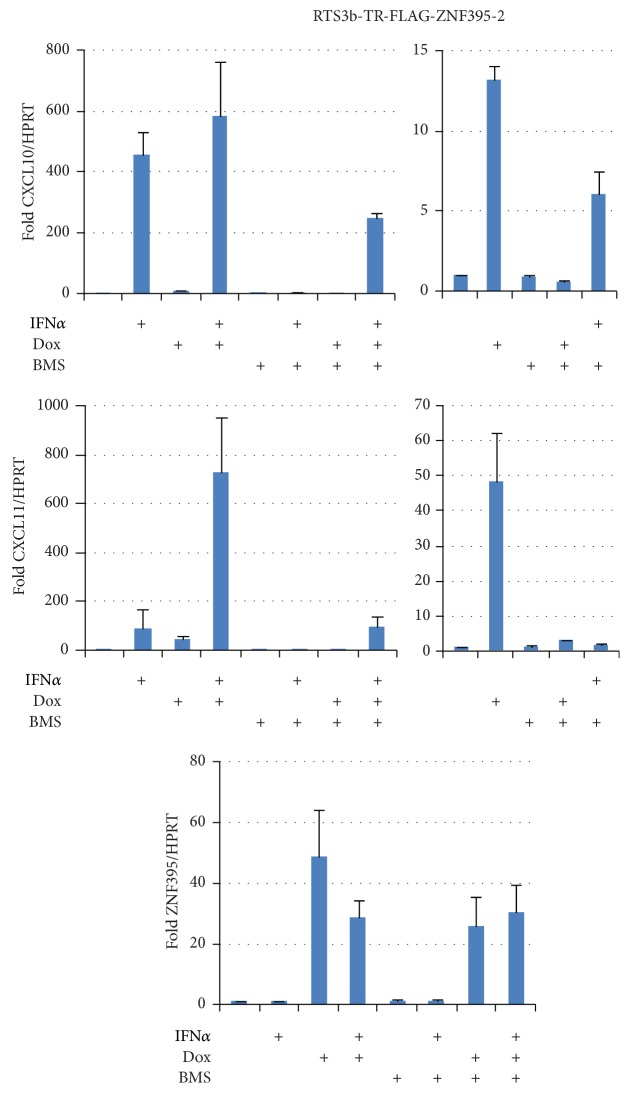
Activation of CXCL10 and CXCL11 expression by ZNF395 requires active IKK. RTS3b-TR-FLAG-ZNF395-2 cells were treated with Dox to induce the expression of ZNF395, with IFN*α* and the IKK*β*-specific inhibitor BMS-345541 in various combinations as indicated in the figure. QRT-PCR was performed to determine the level of transcripts for CXCL10, CXCL11, and ZNF395. After normalization with the house keeping gene HPRT, the values obtained with untreated cells were set as 1 and fold changes upon the different treatments were calculated by Δ-threshold method. The diagrams on the right are provided for a better resolution. The graphs represent the results of three qRT-PCRs with RNA obtained from one experiment. The results have been confirmed with RNA from an independent second experiment (data not shown).

In order to further understand the role of ZNF395 in the expression of CXCL10 and CXCL11 we analyzed the transcription in the presence of BMS-345541, since our previous studies found that the transcriptional activation of the IFIT1 promoter in a luciferase reporter construct by overexpression of ZNF395 required active IKK [12]. BMS-345541 is a highly specific inhibitor of IKK*α* and IKK*β*, the catalytic subunits of IKK [37]. Here, we used the RTS3b-TR-FLAG-ZNF395-2 cell line allowing a higher level of overexpression of ZNF395 by Dox treatment compared to the cell line RTS3b-TR-FLAG-ZNF395-1, used in Figure 2(b) (for details see Section 2) without the need to transfect the cells. As shown in Figure 4, Dox induction resulted in a 13-fold increased CXCL10 expression in the absence of IFN*α*. This activation was completely lost in the presence of BMS-345541. This clearly demonstrates that ZNF395-mediated activation of CXCL10 is not dependent on the IFN-induced transcription factors but on active IKK. In line with the observation shown in Figure 2(b), Dox, that is, elevated expression of ZNF395, only marginally further stimulated the 480-fold IFN*α* induced activation of CXCL10 (Figure 4).

The endogenous CXCL11 promoter was activated 48-fold by overexpression of ZNF395, which again was completely abolished after inhibition of IKK by BMS-345541. Dox-induced ZNF395 expression further increased CXCL11 transcription in response to INF*α* from 92-fold up to 730-fold. This activation was blocked by BMS-345541 as well (Figure 4). These results clearly demonstrate that ZNF395 can stimulate the expression of endogenous CXCL10 and CXCL11, which is independent of IFN*α* but requires active IKK.

### 3.5. ZNF395 Acts through the NF*κ*B Motifs to Stimulate the CXCL10 Promoter

**Figure 5:**
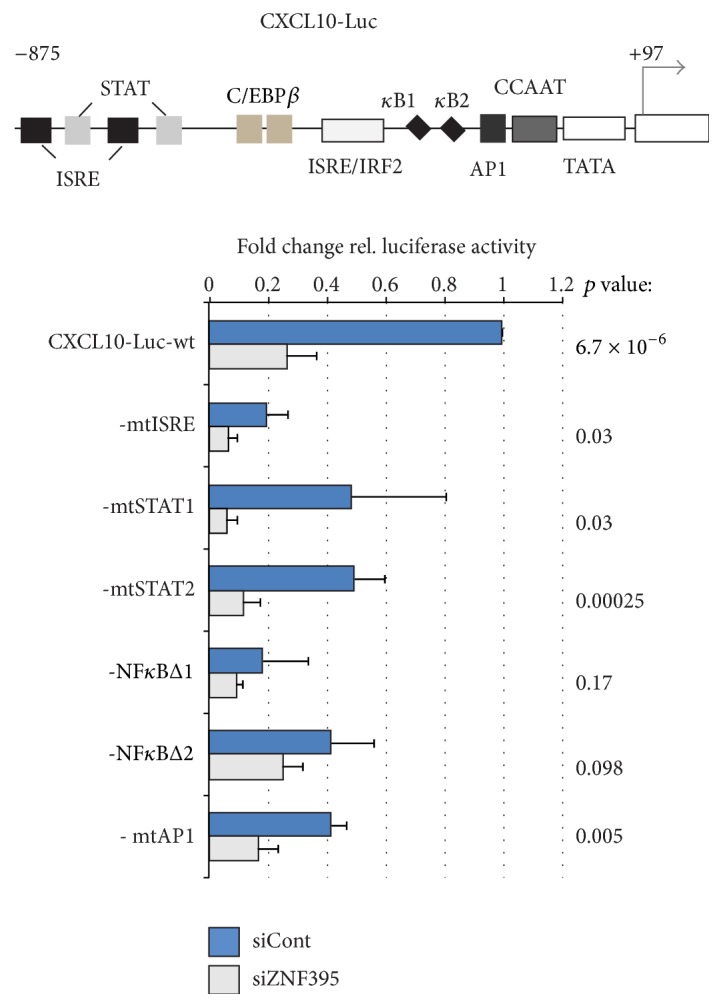
ZNF395 requires intact NF*κ*B motifs to enhance CXCL10 expression. RTS3b cells were cotransfected with the wt CXCL10 promoter-luciferase reporter construct or CXCL10 promoter-luciferase reporter constructs containing mutations within the binding sites of the indicated transcription factors and either 7.5 pmol siRNA against ZNF395 or the same amount of control siRNA. 48 h later the firefly luciferase activity was determined and normalized against the protein concentration of each sample. The relative luciferase activity of the wt CXCL10 promoter in the presence of siControl was set as 1 and the fold activations of the mutated constructs after cotransfection of control siRNA or siRNA against ZNF395 were calculated. The graph represents the average of 6 independent assays. The standard deviations are given. The *p* values calculated by the *t*-test for paired samples are given.

In order to address the contribution of ZNF395 in the promoter activity of CXCL10 more precisely we performed transient transfections with CXCL10 promoter-luciferase reporter constructs. Since the activation of the endogenous CXCL10 promoter by Dox-induced overexpression of ZNF395 was only marginally as shown in Figure 4, we suppressed endogenous ZNF395 by cotransfecting pooled siRNAs against ZNF395 or control siRNA together with the reporter containing the CXCL10 wildtype promoter. Luciferase activity was reduced by 74% when siRNA against ZNF395 was used compared to control siRNA, confirming that ZNF395 is required for the full activity of the CXCL10 promoter (Figure 5). The CXCL10 promoter contains functional binding sites for STAT, NF*κ*B, IRFs, ISGF3, AP1, and C/EBP*β* [38]. To further narrow down the promoter elements through which ZNF395 acts, we used CXCL10 promoter constructs with point mutations in the putative binding sites for NF*κ*B, AP1, ISGF3, and STAT. Again, the ZNF395 specific contribution was assessed by comparing the activities obtained with the mutated promoter constructs after transfection of control siRNA with those obtained after transfection of the specific siRNA leading to the suppression of ZNF395. The mutations within the ISRE element reduced the promoter-activity by 80% compared to the wt promoter in siControl transfected cells. After transfection of siZNF395 the activity dropped by another 30%. Similarly, as a result of the mutations of both STAT motifs (STAT#1, STAT#2) and the AP1 site, respectively, the promoter activity decreased by about 50%, and, again, the suppression of ZNF395 led to a clear further drop of activity. The lower activities with these mutated promoter constructs as a result of the suppression of ZNF395 were significant. The observation that the suppression of ZNF395 further significantly reduced the promoter activities independent of the mutations within ISRE, STAT, and AP1 motif, indicates that endogenous ZNF395 still activates the promoter even in the absence of these factors. In agreement with previous publications, also the mutation of either of the two NF*κ*B motifs had a drastic effect on the CXCL10 promoter. Although the suppression of ZNF395 slightly further reduced the luciferase activities of the NF*κ*B mutated CXCL10 promoters, these effects turned out to be not significant (Figure 5). From this we conclude that ZNF395 requires the intact NF*κ*B motifs to stimulate CXCL10 transcription.

### 3.6. ZNF395 Contains at Least Two IKK-Dependent Degradation Motifs

**Figure 6:**
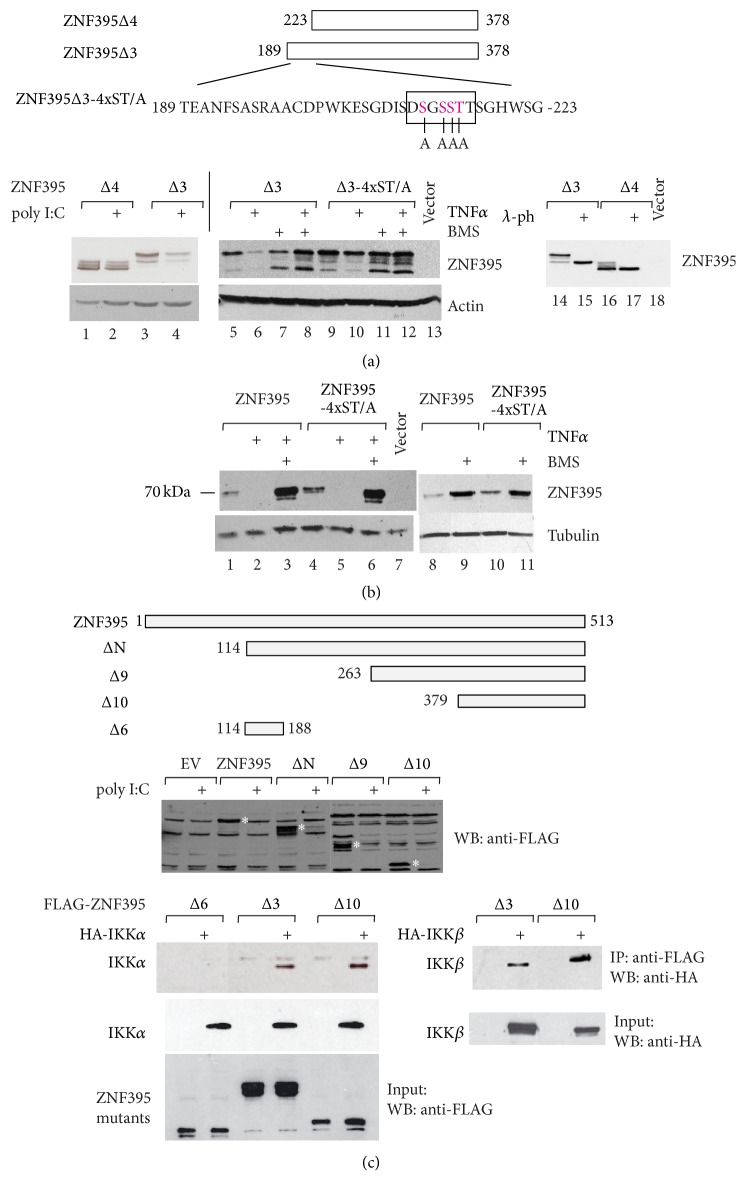
ZNF395 contains at least two independent IKK-dependent degradation motifs. (a) WB with extracts from RTS3b cells which have been transfected with vectors expressing two central fragments of ZNF395, Δ3, encoding amino acids 189–378 and Δ4, encoding amino acids 223–378, or Δ3 with mutations within the putative phosphodegron (lanes 9–12) as indicated in the figure. The cells were treated with poly I:C (lanes 2, 4), TNF*α* (lanes 6, 10), BMS-345541 (lanes 7, 11), or BMS-345541 and TNF*α* (lanes 8 and 12) as indicated. In lanes 15–17 the extracts have been incubated with *λ*-phosphatase (*λ*-ph) prior to analysis. The amino acid sequence from pos. 189–223 is given and the putative phosphodegron motif is indicated as well as mutations present in ZNF395Δ3 4xST/A or ZNF395 4xST/A. (b) RTS3b cells have been transfected with an expression vector for wt ZNF395 or ZNF395 4xST/A containing the mutations shown in (a) and treated with TNF*α* and BMS-345541 as indicated. (c) Expression vectors for ZNF395 and deletion mutants, which are depicted in the figure, were transfected into RTS3b cells which have been incubated with pI:C 24 h prior to harvesting. The WB was developed with the FLAG antibody since the anti-ZNF395 antibody recognizes only an internal fragment of ZNF395 (data not shown). The lower part shows a coimmunoprecipitation with extracts from cells transfected with expression vectors for FLAG-ZNF395, HA-IKK*β*, and HA-IKK*α*. (EV = empty vector). *∗* indicates the position of the full length ZNF395 version.

**Figure 7:**
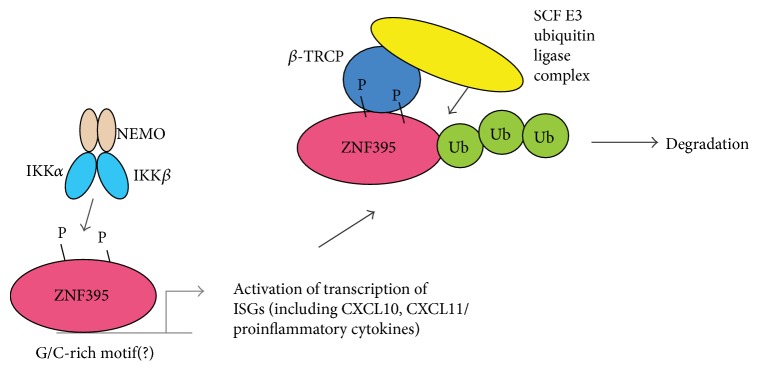
Schematic illustration of the regulation of ZNF395 within the immune response. Active IKK phosphorylates ZNF395, which allows ZNF395 to activate transcription of immune response involved genes. Phosphorylated ZNF395 is a target of the F-box protein *β*-TRCP, which is a subunit of the E3 ubiquitin ligase, the SCF (Skp1-Cullin-F-box) complex. *β*-TRCP directs the conjugation of ubiquitin to ZNF395 leading to its proteasomal degradation, thus keeping the transcriptional activity of ZNF395 low.

Previously we could demonstrate that ZNF395 is a highly instable protein and that the inhibition of IKK increased the stability of ZNF395 [12]. Several genome wide screens found ZNF395 as target of the F-box protein *β*-TRCP which directs the conjugation of ubiquitin to its substrate leading to the degradation of the protein by the proteasome [50–53]. *β*-TRCP-target proteins are characterized by a motif, the so-called phosphodegron DSGXX(X)S/T. The WD40 repeats of *β*-TRCP bind to the DpSGXX(X)pS/T diphosphorylated sequence in its substrates and initiate the degradation. The amino acids from positions 211–216 of ZNF395 with the sequence DSGSSTT are similar to such a motif. We addressed the role of these amino acids in the IKK-regulated turnover of ZNF395. We initially used two fragments of ZNF395, encoding either amino acids 189–378 (Δ3), containing the putative phosphodegron motif, or amino acids 223–378 (Δ4), lacking this element, and overexpressed them in RTS3b cells. To enhance IKK activation and, consequently, degradation of ZNF395 the cells were treated with either TNF*α* or poly I:C (pI:C) [12]. A western blot revealed a reduced intensity of the bands corresponding to ZNF395Δ3 while ZNF395Δ4 was not affected by pI:C (Figure 6(a), lanes 1–4), confirming the presence of a degradation motif within the 35 amino acids from position 189–223 of ZNF395. To analyze the contribution of the putative phosphodegron we mutated the three S in positions 212, 214, and 215 and the T in position 216 into A residues. Figure 6(a) reveals the loss of the TNF*α*-induced degradation and the BMS-345541-mediated stabilization of the mutated ZNF395Δ3 4xST/A fragment (Figure 6(a), lanes 5–12) in contrast to ZNF395Δ3-wt. Thus, this motif mediates the IKK-dependent degradation of ZNF395Δ3. The various bands observed with ZNF395Δ3 and Δ4 represent phosphorylated versions since the treatment of the extracts with *λ*-phosphatase resulted in one faster migrating band, respectively (Figure 6(a), lanes 14–17). Thus, ZNF395 is highly phosphorylated in its central part, not only within the putative IKK-motif but also outside. However, when we introduced these mutations into the full length ZNF395 protein, TNF*α* (as well as pI:C, data not shown) was still able to induce the degradation of ZNF395 (Figure 6(b)), indicating that there are additional motifs present. This was confirmed with a series of deletion mutants covering the entire ZNF395 protein. PI:C (as well as TNF*α*, data not shown) induced the degradation of all fragments. The degradation of ZNF395Δ10, encoding the C-terminal 133 amino acids illustrates that there is one additional sequence mediating IKK-dependent destabilization present in ZNF395 (Figure 6(c)). Previously, we could show that ZNF395 binds to IKK*β* and IKK*α* [12]. Figure 6(c) confirms that, in addition to ZNF395Δ3, also ZNF395Δ10 coprecipitated IKK*β* and IKK*α*. Thus, beyond the canonical *β*-TRCP-dependent phosphodegron motif within its central part ZNF395 has at least one additional degradation motif within the C-terminal part. Taken together, our results suggest that IKK is the protein kinase that regulates ubiquitin-dependent degradation of ZNF395 and that is crucial for activation of transcription by ZNF395 (summarized in Figure 7). We confirm by mutational analysis the functionality of a canonical phosphodegron in the central part of ZNF395.

## 4. Discussion

Here, we report that the endogenous level of ZNF395 determines the IFN-mediated induction of a subset of ISGs including CXCL10 and CXCL11. We visualized the impact of ZNF395 on the expression of these ISGs in the keratinocyte cell line RTS3b in which we modulated the level of ZNF395 over a wide range by siRNA-mediated suppression and by Dox-induced overexpression. The functional relevance of the contribution of ZNF395 to the IFN*α*-mediated control of specific well characterized antiviral ISGs, we have identified here, is supported by several reports correlating reduced ZNF395 expression in patient in the context of an infection with HIV1, CMV, and EBV [29–31]. Our results suggest that ZNF395 plays an important role in the modulation of the antiviral innate immune response.

We confirm the relevance of ZNF395 for the transcription of CXCL10 and CXCL11 in primary keratinocytes. In addition, ZNF395 not only contributes to the IFN*α*- but also to the IFN*γ*-mediated induction of CXCL10 in skin fibroblasts representing the main cell source of this chemokine in the skin [49]. We cannot explain why we did not detect any contribution of ZNF395 to the expression of CXCL11 in HFF with our assay. In contrast to keratinocytes, HFF reveal a higher level of induction of CXCL11 by IFN*α* and IFN*γ*. Maybe the IFN-regulated transcription factors are more efficient or other cofactors are available in HFF making the contribution of ZNF395 dispensable, especially in light of the lower affinity of ZNF395 to the CXCL11 promoter compared to the CXCL10 promoter.

Our results in keratinocytes are in line with the assumption that ZNF395 acts independently of the IFN-induced STAT transcription factors but affects basal promoter activity mainly through the NF*κ*B motifs. Two possibilities concerning the role of ZNF395 can be envisioned. Firstly, the loss of the activating function of ZNF395 is due to the absence of NF*κ*B factors bound at their specific DNA motifs. Here, ZNF395 might be recruited to the CXCL10 promoter via a direct interaction with NF*κ*B subunits. Alternatively, ZNF395 might directly bind to a DNA motif overlapping the NF*κ*B consensus motif. In such a scenario, ZNF395 acts independently of NF*κ*B. Several mutational analysis revealed that the C-clamp DNA binding domains of ZNF395 and its closely related factor SLC2A4RG/GLUT4EF are sufficient for binding to helper site like sequences which have been mapped to C^C^/_G_GC^C^/_G_ [10, 11, 14, 16]. Although the NF*κ*B motifs within the CXCL10 promoter (with the sequences of NF*κ*B1 5′ TGCAACATG**G**GACTTCC**C**CAGGAAC 3′ and of NF*κ*B2 5′ GGAGCAGAG**G**GAAATT**C**CGTAACTT 3′, the underlined nucleotide is mutated) do not contain such a motif, stretches of C/G are present, which have been mutated to A or T nucleotides in the CXCL10 promoter construct we used here [38]. It may be feasible that these bases are involved in a direct DNA binding by ZNF395. The CXCL11 promoter also has one NF*κ*B site [54]. We previously revealed a contribution of ZNF395 to the expression of other proinflammatory factors. ZNF395 was found to be required for the maximal induction of the proinflammatory cytokines IL-1*β*, IL-6, IL-8, and LIF under hypoxia [30]. These genes carry NF*κ*B sites within their regulatory regions as well. It will be tempting to analyze the role of NF*κ*B recognition motifs in the ZNF395-mediated activation of these factors.

Strikingly, our results support the connection of ZNF395 with the NF*κ*B signaling pathway. IKK enables ZNF395 to activate transcription and enhances its turn-over to limit its activity. The observation reported by Loveless et al. that ZNF395 was stabilized by MLN4924, an inhibitor of the cullin-RING E3 ubiquitin ligases, including *β*-TRCP, demonstrates that *β*-TRCP is responsible for the ubiquitin-dependent degradation of ZNF395 [51]. Our observation that the inhibition of IKK by BMS-345541 alleviates the degradation of ZNF395 shows that IKK is the responsible kinase phosphorylating ZNF395 and creating binding motifs for *β*-TRCP. ZNF395 encodes more than one independent acting IKK and *β*-TRCP dependent degradation motif. Beyond the one in the central part, whose functionality we demonstrated here, there is at least one additional degradation motif present in the C-terminal 133 amino acids, although no canonical phosphodegron motif is obvious. It has been observed that several* bona fideβ*-TRCP substrates contain highly degenerated or noncanonical degrons, for instance, DSG/DDG/EEG/SSGXXS/E/D motifs [55]. IKK not only accelerates the degradation of ZNF395 but also stimulates its transcriptional transactivation capacity, which is reminiscent to the IKK-dependent regulation of the NF*κ*B subunit p65. Phosphorylation of p65 induces a conformational change which impacts p65 ubiquitination, stability as well as protein-protein interactions. In addition, the phosphorylation of p65 within its transactivation domain leads to enhanced recruitment of CBP/p300 [56, 57]. The mechanisms as well as the residues of ZNF395 which mediate IKK-dependent transcriptional activation are completely unknown. Nevertheless, our data place ZNF395 among the factors that mediate NF*κ*B-independent functions of IKK [56]. Several reports suggested the existence of an IKK-dependent, NF*κ*B independent mechanism to activate CXCL10 expression. IKK was found to be required for the release of CXCR3 ligands from IFN*γ* stimulated bronchial epithelial cells, independent of NF*κ*B activation. The release of CXCL10 was very sensitive to inhibition of IKK*β*. It has been shown that I*κ*B*α* did not undergo phosphorylation and NF*κ*B was not activated in these cells after stimulation with IFN*γ* [58]. Moreover, IKK was found to play an important role in regulating several activities of type I IFN. Inhibition of IKK suppressed a subset of ISGs in human glioma cells [59].

The CXCL10 and CXCL11 promoters seem to differ in their sensitivity towards ZNF395. The suppression of ZNF395 reduced the IFN*α*-mediated activation of both promoters in keratinocytes (Figures 3(a) and 3(b)), demonstrating their dependence on ZNF395. However, the overexpression of ZNF395 was less effective on the basal as well as on IFN-induced expression of CXCL10, indicating that the CXCL10 promoter reached saturation regarding ZNF395 function. In contrast, ZNF395 overexpression efficiently stimulated the induction of CXCL11, with and without IFN*α*. Thus, ZNF395 may have a higher affinity to the CXCL10 promoter and lower affinity to the CXCL11 promoter, which therefore requires high ZNF395 level for efficient activation. The regulation of the expression of the two CXCR3 ligands in a concentration dependent manner by ZNF395 may also have biological consequences. Despite binding to the same receptor, CXCL10 and CXCL11 were shown to stimulate distinct intracellular signaling pathways. CXCL9 and CXCL10 are so-called driver chemokines that support inflammation. CXCL10 induces Th1/Th17 cells to promote inflammation. In contrast, CXCL11 binding to the CXCR3 results in the development of IL-10 high T regulatory 1 subsets, leading to dampening of inflammation [4]. Taken into account that CXCL11 has higher affinity to the CXCR3, our findings presented here are in line with the model that high level of ZNF395 elevates the amount of CXCL11 thus dampening inflammation while low ZNF395 level might mainly support inflammation via inducing CXCL10.

CXCL10 expression by keratinocytes drives vitiligo pathogenesis through the recruitment of autoreactive CD8^+^ T cells to the epidermis. Vitiligo is an autoimmune disease of the skin that leads to depigmentation. It is initiated by autoimmune T cell mediated killing of melanocytes. These autoreactive T cells are homed to the skin by CXCL10 produced from keratinocytes to kill the melanocytes [7]. In the skin the CXCR3 axis is also involved in the remodeling of wound healing. Wounded keratinocytes express mainly CXCL11, which promotes migration of keratinocytes but inhibits migration of fibroblasts; both effects are mediated via CXCR3 signaling. The disruption of this signaling pathway was shown to delay homeostasis and lead to dystrophic scarring [6]. Regarding the functional role of CXCL10 and CXCL11 expressed from keratinocytes in the pathophysiology ZNF395 may have a profound effect in such processes by modulating the expression of these chemokines.

## 5. Conclusion

Our results demonstrate that the barely characterized transcription factor ZNF395 is required for the maximal IFN-mediated induction of a subset of ISGs. ZNF395 acts independently of IFN. As depicted in Figure 7, the transcriptional activity of ZNF395 is controlled by IKK, which simultaneously enhances the degradation to keep the activity of ZNF395 low. Our results suggest that ZNF395 is a part of the innate immune response and may thus play a role in health and disease.

## Figures and Tables

**Table 1:** Primer for qRT-PCR used in this study.

Gene name	5′primer	3′primer
ZNF395	CGAAAAAAGAAAGAACTCTGTG	CTGTGTCCCCCCAGATGGAG
HPRT	TGACACTGGCAAAAACAATGCA	GGTCCTTTTCACCAGCAAGCT
HERC5	CTGGCACTGTTTAAGAAAC	TCAGCCAAATCCTCTG
TRIM22	GGTTGAGGGGATCGTCAGTA	TTGGAAACAGATTTTGGCTTC
CXCL10	CCAATTTTGTCCACGTGTTG	TTCTTGATGGCCTTCGATTC
CXCL11	AGAGGACGCTGTCTTTGCAT	TGGGATTTAGGCATCGTTGT
MX2	TCTAAGGCCCACAAGCCTTG	CAGTTTCAGCACCAGCGGACACCT

The primers to amplify IFIT1 and IFIT2 were described in [12].

**Table 2:** List of known ISGs which depend for their full activation by IFN*α* on ZNF395, identified in RTS3b keratinocytes by microarray.

Fold change	ANOVA *p* value	Gene symbol	Description	Type of IFN
7.75	0.008142	CXCL10	Chemokine (C-X-C motif) ligand 10	I/II
6.52	0.000568	UBE2D1	Ubiquitin-conjugating enzyme E2D 1	I
4.66	0.003393	INHBA	Inhibin, beta A	I
3.09	0.003036	TMEM27	Transmembrane protein 27	II
2.86	0.000032	SYCP2	Synaptonemal complex protein 2	I
2.75	0.007082	CYP4F2	Cytochrome P450, family 4, subfamily F, polypeptide 2, cytochrome P450, family 4, subfamily F, polypeptide 3	III
2.67	0.000225	CXCL11	Chemokine (C-X-C motif) ligand 11	I/II
2.64	0.000495	IFIT2	Interferon-induced protein with tetratricopeptide repeats 2	I/II/III
2.62	0.000714	IFIT1	Interferon-induced protein with tetratricopeptide repeats 1	I/II/III
2.36	0.001185	H19	H19, imprinted maternally expressed transcript (non-protein coding)	II
2.33	0.000555	TRIM22	Tripartite motif containing 22	I/II
2,33	0.0059	C4orf32	Chromosome 4 open reading frame 32	I
2,29	0.001631	CREBL2	cAMP responsive element binding protein-like 2	I
2.29	0.001	CMPK2	Cytidine monophosphate (UMP-CMP) kinase 2, mitochondrial	I
2.23	0.00487	CINP	Cyclin-dependent kinase 2 interacting protein	II
2.22	0.004045	ITGB4	Integrin, beta 4	II
2.19	0.009825	ZNF702P	Zinc finger protein 702, pseudogene	I
2.16	0.005895	HERC5	HECT and RLD domain containing E3 ubiquitin protein ligase 5	I/II
2.13	0.000467	MT1F	Metallothionein 1F	I/II
2.12	0.001274	PEG10	Paternally expressed 10	II
2.1	0.000303	AMIGO2	Adhesion molecule with Ig-like domain 2	II
2.1	0.008103	MX2	Myxovirus (influenza virus) resistance 2 (mouse)	I/II
2.09	0.000294	OPTN	Optineurin	I/II
2.08	0.000117	ISG15	ISG15 ubiquitin-like modifier	I/II/III
2.03	0.000341	ITGB3	Integrin, beta 3 (platelet glycoprotein IIIa, antigen CD61)	I/II

Given are the fold changes (linear) siControl versus siZNF395 and the ANOVA *p* value siControl versus siZNF395. The results are from three replicates. The type of IFN involved in regulation of the respective ISG is derived from the Interferome database (http://www.interferome.org/interferome/home.jspx; v2.01).
